# The Impact of Local Agrobiodiversity and Food Interventions on Cost, Nutritional Adequacy, and Affordability of Women and Children's Diet in Northern Kenya: A Modeling Exercise

**DOI:** 10.3389/fnut.2020.00129

**Published:** 2020-08-13

**Authors:** Jacob Sarfo, Gudrun B. Keding, Julia Boedecker, Elke Pawelzik, Céline Termote

**Affiliations:** ^1^Division Quality of Plant Products, University of Goettingen, Goettingen, Germany; ^2^Bioversity International, Healthy Diets From Sustainable Food Systems, Nairobi, Kenya

**Keywords:** cost of diet, Kenya, micronutrient powder, wild plant species, women, children, affordability

## Abstract

Wild plant species are often excellent sources of micronutrients and have the potential to promote healthy living, yet they are under-exploited. Distribution of micronutrient powders as diet supplements can play an effective role in reducing micronutrient deficiencies among infants and young children. However, assessing their effects in ensuring a nutritious diet at low cost have been limited. This study assessed the impact of including wild plant species and micronutrient powders in modeled optimized lowest-cost diets for women and children in rural Kenya. Market surveys, focus group discussions in six villages and a 24-h dietary intake recall were used to collect data that were subsequently entered in the cost of diet linear programming tool to model lowest-cost nutritious diets for women and children in Turkana County, Kenya. Three wild vegetables, three wild fruits, and micronutrient powder were added to the models to assess their impact on the cost and the nutrient adequacy of the diets. A locally adapted cost optimized nutritious diet without any intervention costs between 50 and 119 Kenyan shillings (KES) daily ($0.5 to $1.2) for children between 6 and 23 months and 173 to 305 KES ($1.8 to $2.9) for women. Addition of the three wild vegetables resulted in cost reductions between 30 and 71% as well as making up for iron and zinc gaps. The micronutrient powder had an insignificant effect on diet cost and filling nutrient gaps. Edible wild plant species, specifically wild vegetables, can reduce diet costs in considerable proportions while filling nutrient gaps year-round. However, affordability of a nutritious diet remains a major challenge in Turkana County, irrespective of the wealth group.

## Introduction

Nearly 800 million people still suffer from hunger in terms of calorie deficiency, about 2 billion people have insufficient micronutrient intake, and overweight and obesity are on the rise, with about 2 billion adults affected worldwide. Out of 667 million children below the age of five, 159, 50, and 41 million are stunted, wasted, and overweight, respectively (1). Kenya features much in this malnutrition scourge with typical diets in most households lacking iron, vitamin A and zinc micronutrients (2, 3). Turkana County (Northern Kenya) is characterized by arid and semi-arid conditions with limited agricultural activities (4) which exacerbate food and nutrition insecurity (5). Also, more than half (62%) of the population in Turkana lives in absolute poverty—below $1.25/day (6), and therefore, access to a nutritious diet is difficult even in the face of availability (7).

Meanwhile, the Kenyan National Nutrition Action Plan 2012–2017 emphasized on the essence of early child and good maternal nutrition as powerful and protective mechanisms for tackling malnutrition and seeking the well-being of women during pre-conception, pregnancy, and lactating periods as well as their children (8). Therefore, considering the high poverty levels and its correlation with malnutrition, the reduction of diet cost is essential for households to access nutritionally adequate diets (9).

Agrobiodiversity including wild plant species are undervalued within the agro-food system despite their importance in the global food basket (10, 11). Wild plant species have been documented as an important component of diets, in different regions in Africa: they are used as relishes for daily meals or relied on mostly during dry periods (12). They serve as buffer against malnutrition and contribute to improving food and nutrition security year-round due to their favorable composition of nutrients compared to some cultivated crops (10, 11, 13, 14). Most are drought resistant, grow fast and are abundant during periods of food shortage (12). According to Bell-Sheeter (15), intake of these foods promotes healthy living, but with increasing globalization, modernization, habitat loss, and promotion of “modern” crops and foods, wild food production, and intake is often in decline with adverse nutritional effects for the rural poor (13).

On the other hand, nutrition-specific interventions such as distribution of micronutrient powders (MNP) as diet supplement can play an effective role in reducing micronutrient deficiencies among infants and young children (16). It is part of the reasons why Kenya signed up to the scaling up nutrition (SUN) program with the introduction of MNPs to eradicate hidden hunger among children. However, thus far, assessing the implications of such interventions on the cost of a nutritious diet for households has been limited.

The present study, modeled lowest-cost, nutritionally optimized diets making use of the cost of diet (CoD) linear programming tool, developed by Save the Children, UK (17) for women and children in a dry and resource-poor setting, namely Loima sub-County in Turkana County, Kenya. We further estimated the impact of adding local agrobiodiversity (wild plant species) and MNP on the cost and nutritional adequacy of the modeled diets, and assessed which combination (wild plant species alone, MNP alone, both together or none) provides the cheapest cost of diet while meeting nutrient requirements. Finally, the affordability of a lowest-cost nutritious diet for an individual in Turkana was evaluated considering their food expenditure and total income.

## Materials and Methods

### Study Area and Population

Turkana is situated in the north-western region of Kenya with a land area of about 68,183 km^2^ (7). Average temperature is estimated at 30.5°C with erratic rainfall pattern and distribution of 200 mm annually (18). As a result, almost 93% of the land is classified as arid and semi-arid and Turkana is predominantly a pastoralist County (7). Loima sub-county was selected for the study because of its logistical accessibility and because of security reasons. The focus was on all women of reproductive age (15–49 years): non-pregnant/non-lactating; pregnant; and lactating, and children: 6–8; 9–11; and 12–23 months, because they cover the 1,000-day window where adequate nutrition is critical for healthy development (19).

### Study Design and Sample

A combination of cross-sectional surveys and secondary sources of data was employed for this study. First, Loima sub-county was stratified into agro-pastoral and pastoral livelihood zones. Fifteen villages were identified in the agro-pastoral zone and six in the pastoral zone. One village from the agro-pastoral zone and two villages from the pastoral zone were excluded for various reasons including inaccessibility and insecurity. Per zone, three villages were randomly selected proportional to the number of inhabitants in each village. The agro-pastoral zone sample consisted of Nadapal, Lobei, and Kablokor. Lorugum, Namoruputh, and Lokiriama made up the pastoral zone sample. A list of all households with women of reproductive age and at least one child between 6 and 23 months of age was compiled with the help of community health workers for each village. Thirty households per village were randomly selected from these lists for participation in the study. A quantitative 24-h recall tool was applied to the 180 sampled households in September/October 2016. The 24-h recall tool was administered to a woman or caregiver of the young children in each household. The dietary assessment data for both women and young children were used to validate the data on dietary habits collected through focus group discussions. The 2016 household and economic approach (HEA) study performed by Save the Children–Kenya (20) in Turkana was used to gauge the economic status of households in their ability to afford nutritious diets. Data on MNP supplementation for children in Turkana was sourced from the same organization.

### Cost of Diet Tool

This tool was developed by Save the Children UK, and is based on a linear programming mathematical approach that combines locally available foods to compose diets for individuals at the least financial cost while meeting the nutritional requirements of energy, protein, fat, and micronutrients (17). It has been applied in several contexts by Baldi et al. (21); Save the Children UK (22); Geniez et al. (23); Termote et al. (24). The tool is embedded with (i) food composition table data from the FAO's WorldFood Dietary Assessment System, the United States Department of Agriculture (USDA), West Africa, Bangladesh and the cost of diet generic food table, (ii) recommended portion size of foods for individuals, and (iii) recommended energy, macro, and micro nutrients for 237 individuals. To model diets, the tool needs to be uploaded with data from surveys of foods and their prices on local markets, and focus group discussions detailing minimum and maximum frequencies (constraints) of weekly food intake (17).

### Market Survey

Market surveys were conducted in April/May 2016 for the plenty season and July/August 2016 for the lean season in all six villages. Four traders selling mixed food items were selected per village. Survey was conducted in Lodwar market to complement the foods from the six villages. All foods and their corresponding prices were recorded and three different samples of the same price were weighed for each food.

### Focus Group Discussion on Dietary Habits

In July/August 2016, six focus group discussions (FGD) with a total of sixty women were held in the six randomly selected villages to determine culturally accepted dietary habits. Women were selected based on: (a) being members of the sampled households, (b) prepare and serve meals for different household members, and (c) willingness to participate and provide adequate information. With a structured questionnaire, one-on-one discussion was held with each of the women on the frequency of food intake per week. Results were then collated as input for a focus group discussion on food culture. Notes were taken and the discussions were recorded in addition, with oral permission from the women.

### Local Agrobiodiversity and Selection of Wild Plant Species

Data on local agrobiodiversity (ABD) were sourced from an unpublished report by Bioversity International, Kenya (25). In the report, twelve gender-disaggregated FGDs were conducted with 120 participants across the same six randomly selected villages described above, in March/April 2016. Participants were selected based on: (a) their knowledge on agrobiodiversity, (b) having resided at least 1 year in the village, and (c) their availability and willingness to participate and contribute. The participants were asked to recall all foods they know that are produced, bought, sold, and consumed within their community. Using the agrobiodiversity 4-cell methodology developed by Bioversity International, enumerators listed all plant and animal species cited by the participants, and documented their corresponding parts used, preparation and consumption methods and seasonal availability.

Through google scholar search, we used the names of the species to search for peer reviewed articles on nutrient analysis of the wild plant foods in Africa and across the globe. After the search, we were able to compile nutrient composition of 17 wild fruits and 11 wild vegetables present in the ABD list. The nutrient composition included energy, protein, fat, calcium, iron, zinc, thiamine, riboflavin, niacin, magnesium, vitamin C, and A. Compilation was based on 100 g edible portion of fresh weight. Bioavailability rates of 0.48 and 0.05 in the cost of diet tool were used to convert calcium and iron amounts, respectively, into absorbed quantities. β-carotene amounts were converted into retinol equivalent with the ratio 1:12 as used by Termote et al. (24). Three fruits and three vegetables were then selected based on their: (a) micronutrient content especially iron and zinc, (b) availability throughout the seasons, especially lean season, and (c) acceptability across sexes. The selected fruits include *Sterculia africana* (African-star chestnut): a fruit mostly found in arid environments and the side-lines of woodlands. It is berry with yellowish hairs and is pounded and sieved before consumption (26); *Berchemia discolor* (wild almond): it grows well in arid and semi-arid lands (27). The fruits are small drupes, and when ripened, they turn yellow and have a sweet and pleasant smell (28). They are eaten either ripe or unripe (27); and *Grewia tenax* (white crossberry): which is very adaptive to regions with arid conditions and can be intercropped without causing adverse effects (29). The fruit can be eaten raw, made into juice and dried and pounded and eaten with milk or fat (27). The selected vegetables are *Amaranthus hybridus* (amaranth): this plant traces its origin from Central America but became naturalized in Kenya (27). It is commonly found as weed in cultivated fields or barnyards (30). Leaves are used as vegetables, and in western Kenya, they are specifically cultivated in home gardens (27). *Solanum americanum* (American nightshade): it is also known as the American black nightshade which traces its origin from South America and Cuba, and thrives in temperatures above 20°C (31). Leaves and young shoots are used as vegetables, similar to amaranth, and form part of most diets in African homes (32); and *Celosia argentea* (celosia): it is very adaptive to most soils and climates and able to resist drought and heat with few issues of pests and diseases (33). Leaves and flowers are used as vegetables and used for making sauces and soups (33, 34). Table 1 provides the nutritional values of these six species.

**Table 1:** Nutrient composition per 100 g of edible portion of six wild plant foods from the local agrobiodiversity in Turkana selected for inclusion in the models.

**Specie name**	**English name**	**Energy (kcal)**	**Protein (g)**	**Fat (g)**	**Absorbed Ca (mg)^a^**	**Absorbed Fe (mg)^b^**	**Zinc (mg)**	**Thiamine (mg)**	**Riboflavin (mg)**	**Niacin (mg)**	**Magnesium (mg)**	**Vitamin C (mg)**	**Retinol equivalent (μg)^c^**
*Amaranthus hybridus*^d^	Amaranth	80.31	6.30	0.50	265.44	0.55	5.08	0.45	0.70	0.25	329.00	11.16	436.67
*Solanum americanum*^d^	American nightshade	50.33	1.61	2.86	337.21	4.90	1.47	n/a	n/a	n/a	30.01	n/a	n/a
*Celosia argentea^d^*	Celosia	n/a	4.71	1.00	77.92	0.70	6.61	n/a	n/a	n/a	36.14	n/a	n/a
*Sterculia africana*^e^	African-star chestnut	287.52	23.83	6.55	46.67	0.63	5.50	n/a	n/a	n/a	172.77	4.85	n/a
*Berchemia discolor*^e^	Wild almond	266.20	0.99	2.80	9.00	8.75	0.89	n/a	n/a	n/a	1,250.00	59.43	33.71
*Grewia tenax*^e^	White cross berry	322.68	4.50	6.80	900.00	6.25	1.65	n/a	n/a	n/a	1,250.00	161.09	116.67

a *Calcium conversion factor = 0.48, n/a = not available in literature*.

b *Iron conversion factor = 0.05*.

c *β-carotene: retinol equivalent conversion rate = 1:12*.

d *Vegetable*.

e *Fruit*.

*Compiled from multiple sources: (27, 30, 35–38)*.

During the FGDs on dietary habits, the women were asked to determine weekly intake of wild plant foods. The maximum and minimum frequencies of intake per week were identified as three times and one time, respectively. Considering the opportunity cost associated with collecting and preparing the wild foods, market prices of three fruits namely berries, guava, and mango were averaged and used as opportunity cost factor for the wild fruits. For the wild vegetables, market prices of kales, jute mallow leaves, and cowpea leaves were averaged and used to represent the opportunity cost factor. The prices were KES 7.7 for fruits and KES 7.8 ($0.1) for vegetables per 100 g, edible fresh weight.

### Micronutrient Powder

In 2015, Save the Children—Kenya supported implementation of MNP food supplementation program for small children in Turkana County. Young children were targeted because of their vulnerabilities to micronutrient deficiencies. The formulation of the MNP (see Table 2) was based on the national MNP formulation guidelines of the ministry of health in Kenya, which equals the daily recommended nutrient intake (RNI) of micronutrients for children aged 6–59 months. Each child was supplied with 15 sachets per month for at least 6 months (39). Therefore, the minimum and maximum weekly intake levels were three and four, respectively. Although caregivers had to travel to the nearest health center to obtain the powder, the opportunity cost factor was not considered in this analysis.

**Table 2:** Formulation of the micronutrient powder (MNP) used in the supplementation program.

**Micronutrients**	**Amount per sachet of 1 g for Children (6–59 months)**
Vitamin A μg RE	400
Vitamin D (μg)	5
Vitamin E (mg)	5
Vitamin C (mg)	30
Thiamine (mg)	0.5
Riboflavin (mg)	0.5
Niacin (mg)	6
Vitamin B_6_ (mg)	0.5
Vitamin B_12_ (μg)	0.9
Folate (μg)	150
Iron (mg)	10
Zinc (mg)	4.1
Copper (mg)	0.56
Selenium (μg)	17

*Source: (39)*.

### Wealth Groups and Income Level

The household economy approach (HEA) study was carried out by Save the Children, Kenya (20) between June 2015 and May 2016 to determine the various wealth levels in Turkana and their corresponding annual income. Household sizes were identified as well. HEA data gathered on Loima sub-county were used in the framework of this study. Four wealth groups were identified namely the very poor, poor, middle, and better off. Household size, annual income, and percentage of income spent on food among these groups are shown in Table 3.

**Table 3:** Characteristics of different wealth groups identified in Turkana.

**Wealth group**	**Household size**	**Annual income/individual (KES)**	**Percentage of income spent on food**
Very Poor	7	11,786	68
poor	7	14,286	55
Middle	8	21,875	50
Better off	13	21,154	38
Average	8.8	17,275	52.8

*$1 = 103.55 KES (November, 2016)*.

*Source: Modified after (20)*.

### Data Management and Analysis

**Figure 1:**
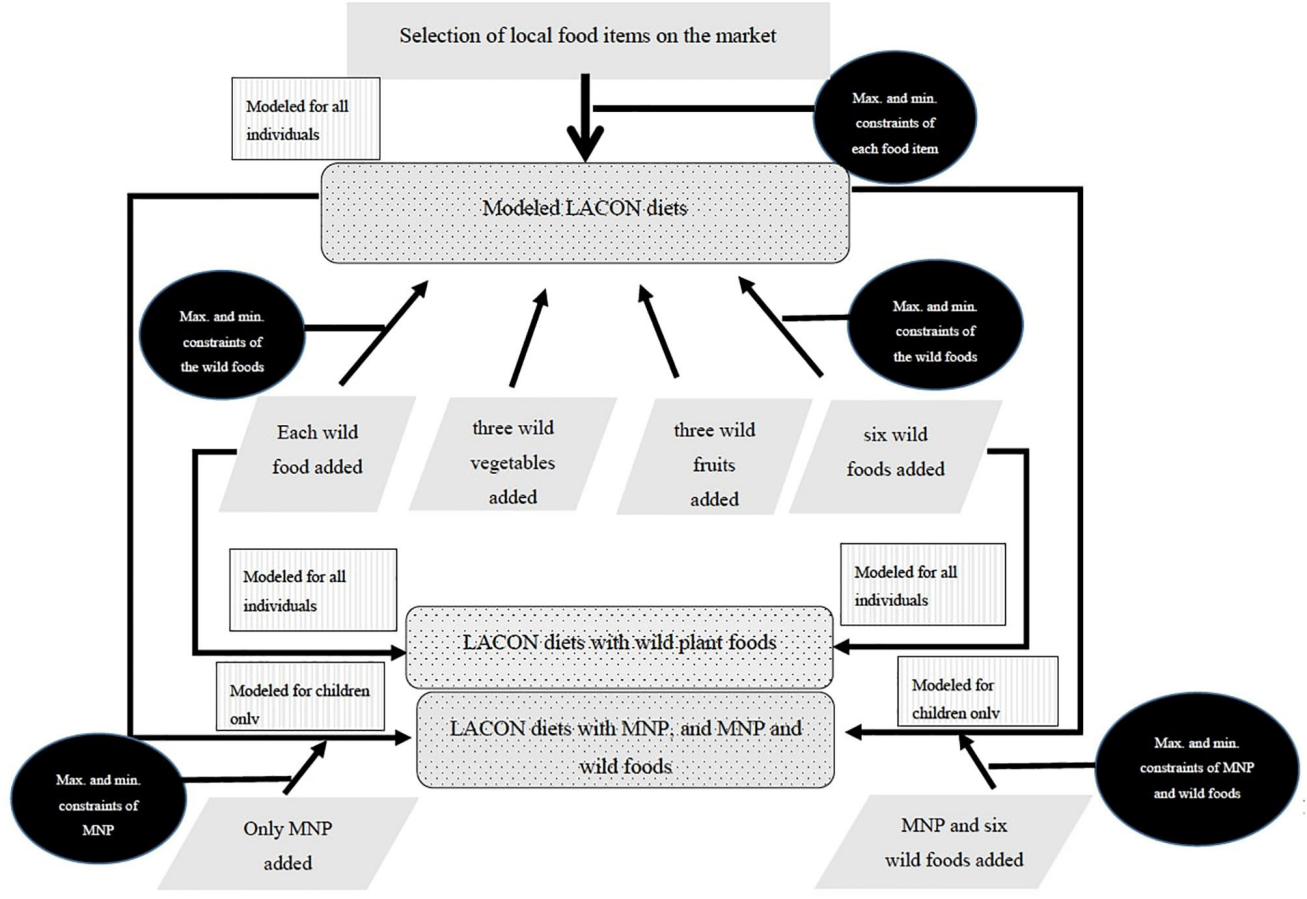
Flow chart depicting how locally adapted cost optimized nutritious (LACON) diets were modeled for women and children in Turkana with and without the inclusion of wild foods and/or micronutrient powder (MNP).

**Figure 2:**
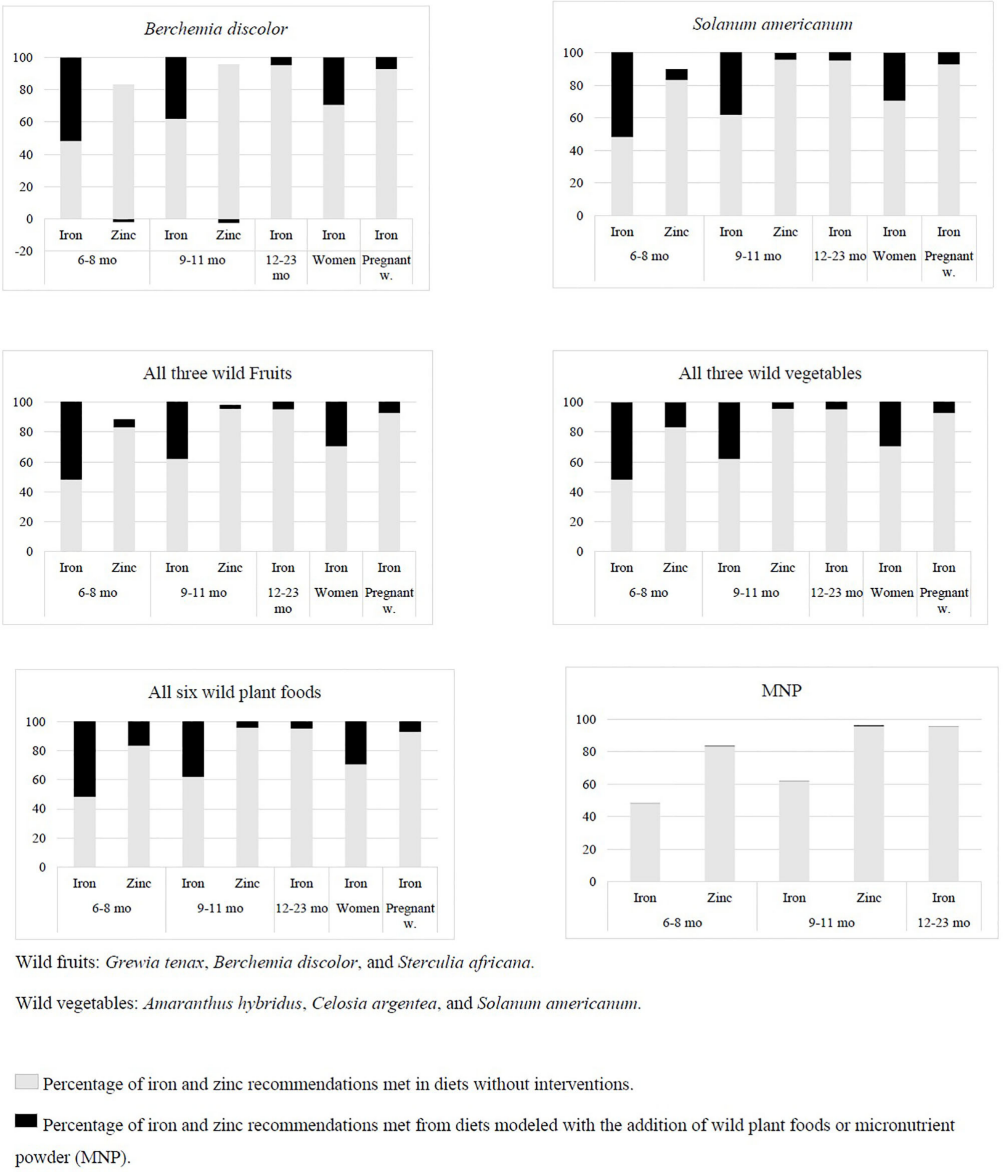
Average percentage of iron and zinc recommendations met without and with wild plant foods and micronutrient powder for children between 6–8 months, 9–11 months, and 12–23 and non-pregnant/non-lactating and pregnant women throughout the year.

The cost of diet tool version 2.2.86 was employed for modeling diets. First, the weights and prices of the foods were directly entered into the tool, which estimated the average prices of the foods per 100 g of edible portion. Minimum and maximum constraints of food intake per week were generated by the tool using the FGD data. The 24-h recall assessment was then used to calculate the number of times food was consumed across households. Minimum and maximum constraints of food consumption were computed at the 25th and 75th percentiles, respectively. The constraints were used to corroborate the constraints from the FGD. Where there were differences between the two datasets, the 24-h recall was preferred as it represented actual dietary intake at the household level. Default recommended portion size of food intake embedded in the tool was used (17). The CoD software was used to simulate locally adapted cost optimized nutritious (LACON) diets according to nutritional recommendations for the different categories of women and children for both plenty seasons which usually occur in March–May and October–December, as well as lean seasons from January–February and June–September in Turkana. LACON diets were first modeled without wild foods or MNPs. Subsequently, the selected wild plant foods including estimated market prices and weekly levels of intake were entered into the CoD tool one-by-one; all three wild vegetables together; all three wild fruits together; and all the six wild foods together to model LACON diets under different agrobiodiversity scenarios. Lastly, the LACON diet was modeled with the MNP and, subsequently, modeled with the MNP and the six wild foods together for the children (Figure 1). All the different intervention scenarios were compared with the no-intervention scenario and a meaningful cost reduction level was set at ≥25% for both seasons. Because there were no significant differences in cost reduction and/or nutrient contributions between seasons, results shown in Table 4 and Figure 2 represent average percentage of cost reductions and nutrient recommendations met, across seasons.

**Table 4:** Daily diet cost across two seasons for children: between 6–8 months, 9–11 months, and 12–23 months and women: non-pregnant/non-lactating, pregnant, and lactating and the average percentage reduction in daily diet costs based on the inclusion of wild plant foods and micronutrient powder (MNP) year-round.

			**Wild fruits**				**Wild vegetables**					
**Individuals**	**Diet cost in plenty season^f^ (KES)**	**Diet cost in lean season^g^ (KES)**	***Grewia tenax***	***Berchemia discolor***	***Sterculia africana***	**All wild fruits**	***Amarant hus hybridus***	***Celosia argentea***	***Solanum americanum***	**All wild vegetables**	**All wild plant foods**	**MNP**
6–8 months	50.0	59.4	−0.3	0.0	−0.2	−27.2^¶^	10.9	20.2	3.0	−71.4^¶^	−70.3^¶^	0.00
9–11 months	65.5	82.3	−19.0	−12.0	−19.0	−35.2^¶^	−8.2	−6.7	−27.7^¶^	−66.1^¶^	−66.4^¶^	0.00
12–23 months	101.5	119.0	−65.2^¶^	−56.4^¶^	−33.6^¶^	−69.2^¶^	−62.6^¶^	−60.0^¶^	−66.0^¶^	−67.6^¶^	−68.9^¶^	−0.6
Women	173.1	226.3	−26.2^¶^	−30.3^¶^	−10.5	−16.3	−8.7	3.9	−28.6^¶^	−29.8^¶^	−22.8	
Pregnant women	247.8	304.7	−44.9^¶^	−46.0^¶^	−12.5	−40.5^¶^	−21.2	−34.0^¶^	−46.7^¶^	−45.3^¶^	−36.0^¶^	
Lactating women	210.3	244.9	−31.3^¶^	−32.6^¶^	−2.5	−27.1^¶^	−15.2	−24.8	−33.3^¶^	−31.6^¶^	−22.6	

¶ *Meaningful cost reduction ≥25%*.

f *March—May and October—December (rain showers)*.

g *January—February and June—September (no rain)*.

*$1 = 103.55 KES (November, 2016)*.

In assessing year-round affordability, the cost of the LACON diet was calculated by averaging the cost of diets for the six individuals (non-pregnant/non-lactating; pregnant; and lactating women, and children: 6–8; 9–11; and 12–23 months) for both seasons. All things being equal, the cost of the LACON diet was assumed to be the equivalent amount to access a nutritious diet by any individual within a household, regardless of age and sex. The assumption was on the basis that diet modeling excluded individuals like men, and the analysis of the income data was per person/individual and not adult male equivalents. The percentage of food expenditure of each wealth group (taken from the HEA 2016) was then used to estimate the affordability of a nutritious diet. The affordability estimation was repeated using the assumption that the entire income per individual could be used for food expenditures.

## Results

### Agrobiodiversity and Food Intake

Bioversity's ABD report presented a total of 123 edible plant species of which 95 were taxonomically identified and 28 remained unidentified. Of the 95 identified species, 34 were cultivated while 61 were considered wild plant species (Supplementary File 1). During the market surveys, we found 56 food items on the market including six animal-sourced foods across seasons. Comparing the list of food items found on the markets with the dietary intake data showed that almost half of the foods (48%) were not found in the diets of the study participants including no fruit intake recorded.

### Cost of Diets With and Without Wild Plant Species

LACON diets without any intervention costed between 50 and 102 KES daily ($0.5 to $1) for children between 6 and 23 months in the plenty season. LACON diets for women costed between 173 and 248 KES ($1.8 to $2.4). In the lean season, where availability of foods was considered to be low, diet cost for children increased by almost 21%. An average increase of 23% was estimated for women. Each of the wild foods added to the standard modeled diets of children between 12 and 23 months had a meaningful cost reduction effect. Cost reductions between 34 and 66% (Table 4) were documented. Only *Solanum americanum* (American nightshade) reduced the diet cost for children between 9 and 11 months at a meaningful proportion, by 28%. For children between 6 and 8 months, there was a negative cost reduction impact, except for *Grewia tenax* (white cross-berry) and *Sterculia Africana* (African-star chestnut). *Solanum americanum, Grewia tenax*, and *Berchemia discolor* (wild almond), reduced the cost at meaningful proportions for women year-round. The three wild vegetables together [*Amaranthus hybridus* (amaranth), *Solanum americanum*, and *Celosia argentea* (celosia)] provided the most meaningful cost reduction potential for all individuals (women and children) throughout the year as shown in Table 4. Their cost reduction potential was between 30 and 71%. The impact of the three wild fruits together (*Grewia tenax, Berchemia discolor*, and *Sterculia Africana)* was below that of the vegetables. Combination of vegetables and fruits added to the models resulted in meaningful reductions in costs for children and pregnant women.

### Effect of Micronutrient Powder on Cost

Surprisingly, the MNP had little or no effect when added to the models for the children. Adding the MNP to the standard LACON diets in Turkana, resulted in a <1% reduction in diet cost for children. Since the MNP made no positive effect, adding MNP plus the six wild foods in the models provided the same results as adding the six wild foods alone (percentage reduction not shown in Table 4).

### Nutrient Profile of Diets

Without interventions (wild foods and/or MNP), iron and zinc deficiencies were found in the modeled diets for children and women, except lactating women. The diets potentially supply between 48 and 95% of the iron requirements needed by children. Zinc deficiency was detected in the diets of children between 6 and 11 months. For non-pregnant/non-lactating and pregnant women, there was a shortfall in their iron supply (Figure 2). In particular, the vegetable *Solanum americanum* bridged the supply gaps except for zinc in the diet of children at the age of 6–8 months. Addition of *Berchemia discolor* to the modeled diets for children between 6 and 11 months, however, even lowered the zinc content by 2%. Zinc remained deficient for children between 6 and 11 months when the three fruits were added to the models. The three vegetables, however, were able to fill the nutrient gaps in the diets of women and children. Diets for children with MNP still revealed iron and zinc deficiencies in the models. MNP improved the iron and zinc contents by <1% (Figure 2).

### Affordability of the Cheapest LACON Diet

**Figure 3:**
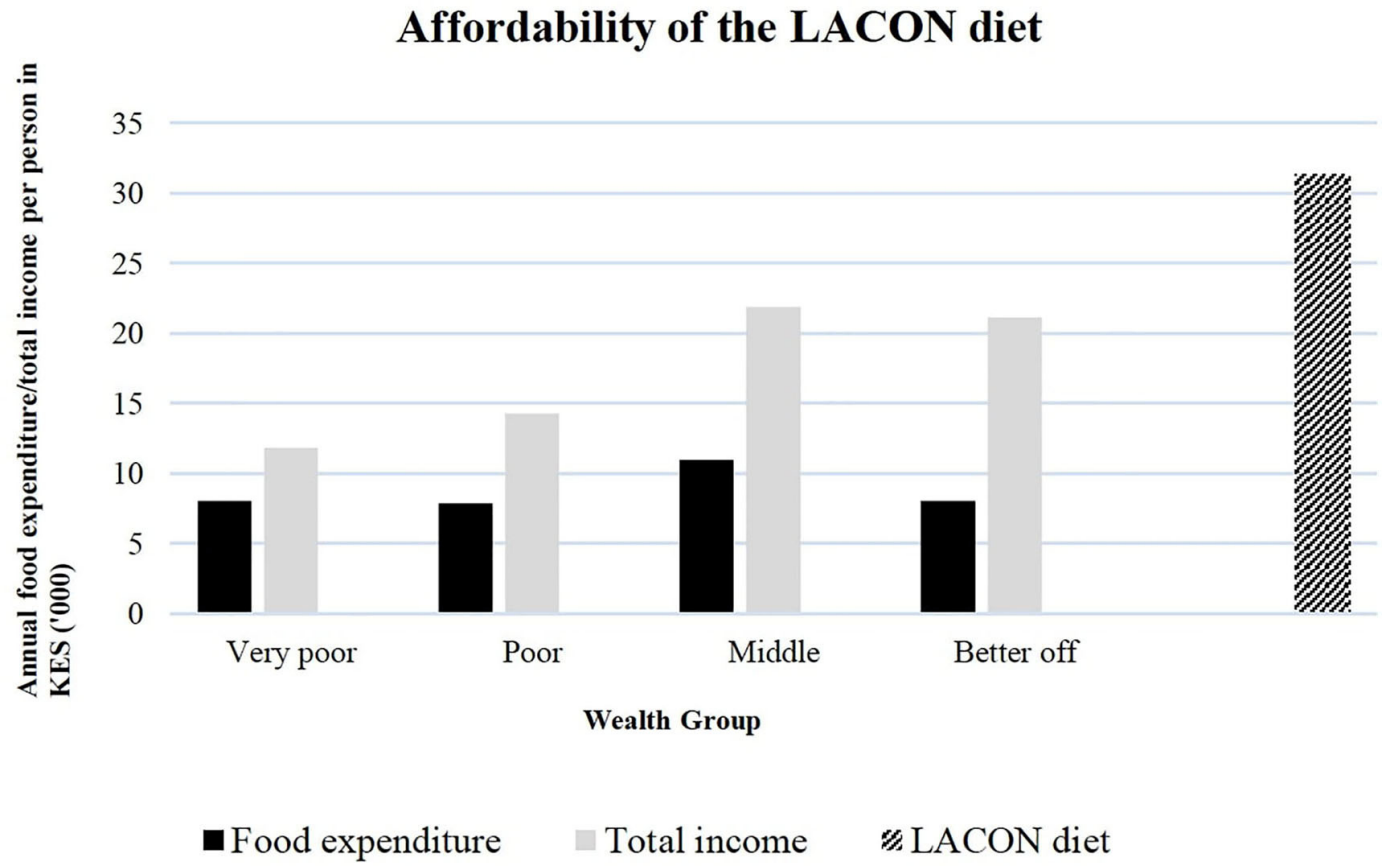
Affordability of the locally adapted cost optimized nutritious (LACON) diet–modeled with three wild vegetables–per person for the various wealth groups in Turkana County, Kenya.

The cheapest LACON diet was provided by adding the three wild vegetables together in the models. Based on the assumption stated earlier on, it would cost approximately 31,295 KES equivalent to $302 annually for any individual within a household to access the nutritionally adequate diet with wild vegetables. Unfortunately, none of the wealth groups could afford this diet taking into account the proportion of income spent on food. Even if the total income would be used for food expenditures, most households would still not be able to afford the LACON diet (Figure 3).

## Discussion

The number of food items (56) identified on the market in Turkana was similar to the number reported by Termote et al. (24) for Baringo district, Kenya. As a result of high food prices, the majority of these foods do not form part of households' diets. Banana was the only fruit identified during the market survey. Other fruit products found were mainly juices, which are considered expensive by households. No fruit intake was recorded during the recall. This is in sharp contrast to the recommended 200 g/capita/day intake by the WHO (40). Consequently, fruit intake is a challenge in Turkana. In western Kenya where the climatic conditions are favorable for fruit production, high fruit intake is also a challenge (41). However, it must be noted that a single 24-h recall was carried out in one season in Turkana and it is thus still possible that fruits are consumed during other periods of the year.

From the focus group discussions, we discovered that households tend to eat more in the plenty season than in the lean season, and modeled diet costs are higher in the lean season. This could be mainly due to higher food prices during that period because of reduced supply. The standard diets of children, and non-pregnant/non-lactating and pregnant women without the interventions, lacked iron and zinc despite the objective of the cost of diet tool to optimize diets to meet recommended macro- and micronutrient intakes. In Mozambique, Frega et al. (42) showed that iron and vitamin B_2_ were the limiting nutrients in their modeled diets. Termote et al. (24) also revealed a lack of iron, zinc, calcium, vitamin B_6_ in CoD modeled diets in Baringo, Kenya. In a study done by Arimond et al. (43) in Burkina Faso, the authors indicated that recommended intakes for iron, vitamin B_2_, B_6_, and B_12_, folate and calcium are difficult to achieve through local food sources. This implies that iron is a primary missing nutrient in diets across countries. Although iron is present in many foods, the absorption rates from diets are usually low, resulting in lower contributions than what the body requires (44, 45). Mostly, diets consumed in developing countries are plant-based and predominantly made up of cereals, legumes, roots and tubers, some vegetables, and fruits. The bioavailability of iron in these foods is estimated to be <10% relative to 15–35% found in meat (44, 45). Iron deficiency causes anemia, and the risk is high among young children, adolescents, and pregnant women. Iron requirements triple for pregnant women relative to the requirements for lactating women. In Africa, 3.7% of maternal deaths are attributable to iron deficiency during pregnancy (44). Like iron, zinc is a limiting nutrient due to low absorption levels caused by fiber inhibitors (46). Therefore, cheap but nutritious plant-based diets could be diversified with animal-sourced foods such as meat to increase iron and zinc availability in diets.

Comparing edible plant species within various sites in Kenya, the number identified in this study (123 species) is lower than what was found (177 species) by Termote et al. (24). However, higher than the number of species found in western Kenya (59 species) and Nyanza Province (49 species) as identified by Ng'endo et al. (47) and Remans et al. (48), respectively. The identified wild plant species provide an extensive list of opportunities that can be exploited to ensure food and nutrition security in Turkana and beyond. So far in Kenya, wild foods have a great potential to reduce diet cost for children 12–23 months and women as well as to make up for dietary gaps as demonstrated in the present study and the study by Termote et al. (24). However, the exact wild foods or combinations of wild foods at the basis of these effects are location-specific. Unsustainable harvesting practices put species at risk which in turn affect the ecosystem (49). Hence, efforts to domesticate these plant species in farmlands and kitchen gardens must be intensified especially for regions where they are known to thrive even at the harshest climate conditions. Home gardening has a positive effect in addressing food insecurity and malnutrition (50). Already in certain parts of Kenya like the Bondo district, some of these species including amaranth and black nightshade, which are considered as wild in Turkana, are cultivated on farmlands and kitchen gardens. They are considered as readily available nutritious indigenous foods (51) which can be cultivated in addition to staple crops including sorghum, millet, beans, and some vegetables (jute mallow leaves, kales, cowpea leaves). The ability of wild vegetables to reduce diet cost at huge proportions across the board showcases the potential of local agrobiodiversity in sustaining households, particularly the resource-poor.

MNPs, as distributed (3 to 4 sachets per child per week) in Turkana, had surprisingly very little effect on the children's diet cost and nutrient adequacy. When the weekly intake for MNP was adjusted to seven (one sachet per day), the impact remained the same. This is in contrast to a study done by Baldi et al. (21) in Indonesia where a child's diet cost was reduced by $0.12 with addition of a sachet of MNP three times per week. Therefore, it is assumed that one gram of MNP assigned daily might be small relative to the recommended total food intake in the hypothetical diets to trigger a significant positive effect. Moreover, the extent of an intervention's impact on modeled diets depends on the nutritional values of the locally available foods and their respective intake patterns fed into the CoD tool. Home-based fortification of diets with MNP is said to be an effective intervention to minimize iron deficiency in young children (52). However, in Turkana, the addition of MNP provided adverse results on hypothetically modeled diets. This might be due to low iron and zinc contents provided by the MNP especially when formulation variations between sachets can go up to 20% (53). Schauer et al. (54) highlighted that, MNPs were often introduced without proper baseline studies to understand the area-specific nutritional challenges and without review of existing policies and programs within communities. The MNP formulation and distribution in Turkana were based on a generic approach—following the Kenya national MNP formulation guidelines, while Kenya is a huge and very diverse country in terms of ecological zones and food habits. In Columbia, MNP interventions proved to be ineffective at reducing childhood anemia because of the inefficacy of the product vis-à-vis the target individuals. Children from 12 months were identified as already too old with adequate levels of hemoglobin for MNP to be effective as a nutritional intervention (55).

Affordability of a nutritious diet is a challenge faced by most households in Turkana. Likewise, Geniez et al. (23) highlighted that many households in Nepal were unable to access nutritious diets as a result of financial constraints. Meanwhile, the ability of households to afford diets that meet nutritional requirements is inversely correlated with the prevalence of stunting and underweight among children under 5 years (21). For Turkana households to be able to afford nutritious diets, alternative income sources are needed. This could be, among others, (i) home-based processing of milk by the women for extra income, as milk found on the market were all industrially processed; (ii) home gardening for home consumption and sale; (iii) sustainable harvesting options of wild plant foods for consumption and sale with a medium to long term plan of domestication on farmlands and gardens. According to Bharucha and Pretty (10) and Shumsky et al. (56), edible wild species offer a source of livelihood to most small-holder households characterized by food insecurity, no off-farm income source, and weak asset base.

LACON diets modeled with wild vegetables demonstrate that wild plant foods provide good sources of micronutrients coupled with optimized costs through which vulnerable individuals can improve their food and nutrition security.

## Study Limitations

This study was limited by the fact that a single 24-h dietary recall was carried out for only one season. A single recall in only one season does not provide accurate results of the usual food and drinks intake year-round. Two or three dietary recalls are usually recommended in addition to capturing seasonal variations of food and drink intake (57).

According to Ng'endo et al. (47), indigenous leafy vegetables provide cheap micronutrient sources for diets as also shown in this study. However, in general, there are certain limitations to the efficacy of wild plant foods. Low bioavailability of iron and zinc in plant food sources may limit their effectiveness. Also, availability might be a huge challenge if most households solely rely on their supply from the wild or forests, and consequently, posing negative impacts on the environment.

Data on nutritional composition of wild foods are limited, and when available, mostly from other geographical settings (24, 43) other than the study area. This does not provide their precise impact on location-specific basis leading to over or under estimation of their potentials. The study of wild foods' impact on the cost of diet is also limited by their lack of prices on local markets.

## Study Recommendations

Based on the findings of this study, the authors propose the following policy recommendations and areas for further research:

- Fruits and vegetable production and in addition, processing are vital steps to ensuring availability and to some extent accessibility for intake. This study therefore recommends that production and processing of fruits and vegetables, and food in general, must be boosted with technical assistance, new technologies, and inputs in areas where climate conditions are more favorable for further supply to locations hindered by climatic conditions.
- More research is needed into sustainable harvest options and/or domestication to support consumption of wild food species.
- Nutrition education integrating the importance of wild foods to diets and as a livelihood source must be pursued.
- To assess the actual nutritional impact of wild foods instead of approximations, more research is needed in the area of compiling the nutritional composition of these foods. Also, nutritional analysis of wild foods should be paired with proper taxonomic indentification of the wild foods. Further research on identifying their true market values and the environmental impact of wild harvesting is much needed.
- Besides diversifying diets with locally available agrobiodiverse foods, fortification of staple foods with high absorption levels of iron and zinc is an option to address micronutrient deficiencies, especially for households who are unable to afford animal-based foods, to diversify their diets.
- Micronutrient powder formulations should be based on nutrient gaps identified within the locality of interest and take into account absorption levels of the nutrients to be provided. This will improve their efficacy in addressing micronutrient deficiencies.
- Lastly, modeled diets are considered hypothetical diets with certain limitations. Despite the constraints used in the modeling tool to reflect cultural acceptability, the acceptability and feasibility of the modeled diets is not guaranteed. More research is needed into actual dietary patterns and the lowest-cost acceptable dietary adaptations that have potential to improve diet quality.

## Conclusion

The present study compared the impact of two interventions: (a) integrating agrobiodiversity; and (b) a nutrition-specific intervention with micronutrient powder, on the diet of small children and women. It demonstrated that edible wild plant species, specifically wild vegetables, which are a part of agrobiodiversity, contribute to food and nutrition security by reducing diet costs significantly as well as bridging nutrient gaps for various individuals all year-round. However, affordability of the nutritious diets modeled with wild vegetables remains a major challenge irrespective of the wealth level.

## Data Availability Statement

The raw data supporting the conclusions of this article will be made available by the authors, upon reasonable request.

## Ethics Statement

Amref Ethics and Scientific Review Committee in Kenya reviewed and approved the research protocol for this study (ESRC P276/2016). Written or thumb-printed informed consent was obtained from the study participants.

## Author Contributions

JS, JB, GK, and CT were responsible for the conception and design of this study. JS collected and analyzed the data and wrote the first manuscript. JB, GK, EP, and CT reviewed and edited the manuscript. All authors read and approved the manuscript for submission.

## Conflict of Interest

The authors declare that the research was conducted in the absence of any commercial or financial relationships that could be construed as a potential conflict of interest.
