# Supplementary material for: The Impact of Local Agrobiodiversity and Food Interventions on Cost, Nutritional Adequacy, and Affordability of Women and Children's Diet in Northern Kenya: A Modeling Exercise
Source: Front Nutr. 2020 Aug 13;7:129. doi: 10.3389/fnut.2020.00129 (PMC7443573; doi:10.3389/fnut.2020.00129)
Supplement: Supplementary file 1 [file Table_1.DOCX]

*Supplementary file 1: Wild plant foods within the local agrobiodiversity in Turkana*

| Species local name | Family | Species Scientific name&Author | Part used | Type (Annual=1, Perennial=2) |
| --- | --- | --- | --- | --- |
| Abach | Canellaceae | *Warbugia ugandensis* Sprague | Bark | 2 |
| Akeyo/Sagaa | Cleomaceae | *Cleome gynandra L.* | Leaves | 1 |
| Akoporait | Convolvulaceae | *Ipomoea mombassana* Vatke | Leaves | 1 |
| Aloe vera | Xanthorrhoeaceae | *Aloe vera* (L.) Burm.f. | Leaves | 2 |
| Angolekothim | Amaranthaceae | *Celosia argentea* L. | Leaves | 1 |
| Auriengo/Aurengo/Auriongor | Hydnoraceae | *Hydnora abyssinica* A. Br. | Tubers | 2 |
| Dodo | Amaranthaceae | *Amaranthus hybridus* L. | Leaves | 1 |
| Ebei | Zygophyllaceae | *Balanites rotundifolia* (Tiegh.) Blatt. | Fruit | 2 |
| Eburui | Celastraceae | *Gymnosporia senegalensis* | Fruit, leaves | 2 |
| Echoke | Salicaceae | *Flacourtia indica* (Burm.f.) Merr. | Fruit | 2 |
| Edaladala sikin/Ekadala | Cucurbitaceae | *Coccinia grandis* (L.) Voigt | Leaves, Fruits | 2 |
| Edapal | Salvadoraceae | *Dobera glabra* (Forssk.) Juss. ex Poir. | Fruit | 2 |
| Edapal-pus | Euphorbiaceae | *Euphorbia nutans* Lag. | Fruit | 2 |
| Edome | Boraginaceae | *Cordia sinensis* Lam. | Fruit | 2 |
| Eedung | Capparaceae | *Boscia coriacea* Graells | Fruit, Grains | 2 |
| Eerut | Capparaceae | *Maerua decumbens* (Brongn.) DeWolf | Fruit, Grains | 2 |
| Egilae | Leguminosae | *Vatovaea pseudolablab* (Harms) J.B.Gillett | Leaves, Tuber | 2 |
| Ekabekebeke | Combretaceae | *Combretum aculeatum* Vent. | Leaves | 1 |
| Ekadeli | Burseraceae | *Commiphora africana* (A.Rich.) Endl. | Bark | 2 |
| Ekamongo | Apocynaceae | *Leptadenia lancifolia* (Schumach. & Thonn.) Decne. | Leaves | 2 |
| Ekamurae/Ekamuria | Apocynaceae | *Carissa spinarum* L. | Fruit | 2 |
| Ekaye | Primulaceae | *Myrsine africana* L. | Bark, Seeds | 2 |
| **Ekiliton/Lokiliton** | Amaranthaceae | *Amaranthus graecizans* L. | Leaves | 1 |
| Ekumait | Leguminosae | *Acacia senegal* (L.) Willd. | Fruit | 2 |
| Ekwangorom | Burseraceae | *Commiphora schimperi* (O.Bergman) Engl. | Bark | 2 |
| Elamach | Zygophyllaceae | *Balanites pedicellaris* Mildbr. & Schltr. | Grains | 2 |
| Emeyan(Ngameyana) | Rhamnaceae | *Berchemia discolor* (Klotzsch) Hemsl. | Fruit | 2 |
| Emidakan | Rubiaceae | *Bullockia pseudosetiflora* (Bridson) Razafim., Lantz & B.Bremer | Fruit | 2 |
| Emus | Euphorbiaceae | *Euphorbia vaginulata Griseb.* | Whole plant | 2 |
| Eng'ol | Arecaceae | *Hyphaene compressa* H.Wendl. | Fruit, Seeds | 2 |
| Engomo | Malvaceae | *Grewia tenax* (Forssk.) Fiori | Fruit | 2 |
| Eome | Cucurbitaceae | *Cucumis dipsaceus* Ehrenb. ex Spach | Leaves | 1 |
| Eosin-aikeny | Amaranthaceae | *Digera muricata (L.) Mart.* | Leaves | 2 |
| Epat | Malvaceae | *Grewia mollis* Juss. | Fruit | 2 |
| Epoteny/Eputen | Apocynaceae | *Brachystelma johnstonii N.E.Br.* | Tubers | 2 |
| Erau | Passifloraceae | *Secale cereale L.* | Seeds | 2 |
| Eroronit/Eroronyit | Zygophyllaceae | *Balanites aegyptiaca* (L.) Delile | Fruit | 2 |
| Esanyanait | Leguminosae | *Acacia elatior* Brenan | Bark | 2 |
| Esekon | Salvadoraceae | *Salvadora persica* L. | Fruit, Roots | 2 |
| Esikilelee | Apocynaceae | *Calotropis procera* (Aiton) Dryand. | Seeds | 2 |
| Esuguru | Zygophyllacea | *Tribulus cistoides* L. | Leaves | 1 |
| Esungu/Eusong/Eusugu | Rutaceae | *Zanthoxylum chalybeum* Engl. | Seeds | 2 |
| Etete | Malvaceae | *Sterculia africana* (Lour.) Fiori | Fruit | 2 |
| Etopojo | Anacardiaceae | *Lannea triphylla* (Hochst. ex A. Rich.) Engl. | Tubers | 2 |
| Eurumosing | Burseraceae | *Commiphora rostrata* Engl. | Leaves,bark,seeds | 2 |
| Loarakimak/Loarakimale/Larakimak | Passifloraceae | *Adenia volkensii Harms* | Leaves, Seeds | 2 |
| Mapera | Myrtaceae | *Psidium guajava* L. | Fruit | 2 |
| Mkoma | Arecaceae | *Hyphaene coriacea* Gaertn. | Fruit | 2 |
| Mrere | Malvaceae | *Corchorus olitorius* L. | Leaves | 1 |
| Ngachokio | Moraceae | *Ficus sycomorus* L. | Fruit | 2 |
| Ngacholae | Poaceae | *Sorghum bicolor* L. | Tubers | 1 |
| Ngakalalio | Rhamnaceae | *Ziziphus jujuba* Mill. | Fruit | 2 |
| Ngakalio | Malvaceae | *Grewia damine* Gaertn. | Fruit | 2 |
| Ngakima/Ngikaman ngimel | Poaceae | *Pennisetum glaucum* (L.) R.Br. | Grains | 2 |
| Ngalam | Olacaceae | *Ximenia americana* L. | Fruit, Seeds | 2 |
| Ngapedum/r | Leguminosae | *Tamarindus indica* L. | Fruit | 2 |
| Ngapeeto | Leguminosae | *Acacia reficiens* Wawra | Bark | 2 |
| Ngaponga/Ngapongae | Malvaceae | *Grewia villosa* Willd. | Fruit | 2 |
| Ngimaru | Leguminosae | *Indigofera schimperi* Jaub. & Spach | Tubers | 2 |
| Ngitit | Leguminosae | *Acacia tortilis* (Forssk.) Hayne | Fruit, Seeds | 2 |
| Suja | Solanaceae | *Solanum americanum* Mill. | Leaves | 1 |
